# Non-biological factors associated with postpartum depression among women in Shenzhen: a case-control study

**DOI:** 10.3389/fpubh.2024.1417861

**Published:** 2024-09-11

**Authors:** Jiangshan He, Yang Li, Ling Chen, Ying Zhang

**Affiliations:** ^1^Second Clinical College, Guangzhou University of Chinese Medicine, Guangzhou, China; ^2^Eighth Affiliated Hospital, Sun Yet-san University, Shenzhen, China; ^3^Maternity and Children Health Care Hospital of Luohu District, Shenzhen, China; ^4^National Clinical Research Center for Chinese Medicine Cardiology, Xiyuan Hospital, China Academy of Chinese Medical Sciences, Beijing, China

**Keywords:** postpartum depression, China, cultural factor, family relationship, depression

## Abstract

**Background:**

Postpartum depression (PPD) presents a significant public health challenge. While PPD’s impact extends from maternal health to child development, cultural stigma and a lack of public awareness, particularly in developing countries, contribute to its underestimation and under diagnosed. This study investigated the non-biological associated factors for PPD in Shenzhen city due to its unique socioeconomic landscape, where rapid urbanization and migrant influx could uniquely impact maternal mental health. By identifying local PPD determinants, the research aimed to contribute to targeted mental health interventions in the region.

**Method:**

Data were collected from May to December 2019 at the Luohu Maternal and Child Health Medical Center, Shenzhen. Inclusion criteria were postpartum women without psychiatric histories who live within the locality. The Chinese Edinburgh Postnatal Depression Scale was utilized to confirm PPD diagnosis. Participant information including demographics, economic status and postnatal factors were collected via structured questionnaires. Statistical analyses of *t*-tests, Wilcoxon rank-sum tests, chi-square tests, and logistic regression, were performed using SPSS 20.0, with significance set at *p* ≤ 0.05.

**Results:**

The study included 430 healthy mothers and 73 PPD mothers. Several factors were found to significantly influence the onset of PPD (*p* < 0.05): age (OR = 0.921, 95% CI: 0.864–0.981); living with in-laws (OR = 2.133, 95% CI: 1.108–4.106); bottle feeding (OR = 3.757, 95% CI: 1.567–9.006); prenatal depression (OR = 3.515, 95% CI: 1.61–7.675); prenatal anxiety (OR = 6.072, 95% CI: 3.209–11.49); and adverse life events during pregnancy (OR = 3.287, 95% CI: 1.165–9.269). Other factors were not found to have a significant effect.

**Conclusion:**

Our study found that in the developed city of Shenzhen in Southern China, living with in-laws, exclusive bottle feeding, prenatal anxiety, depression, and adverse life events are non-biological associated factors for postpartum depression. The findings emphasize the importance of considering a range of factors when addressing maternal mental health within a specific local regions. It calls for targeted interventions or prevention program that take into considering the specific cultural, social, and individual factors.

## Introduction

Postpartum depression (PPD) is commonly known as postnatal depression. The prevalence of postpartum depression varies from 1.9 to 82.1% in developing countries (1) and from 5.2 to 74.0% in developed countries (2). In China, the prevalence of PPD was reported to be around 15% (3) to 19% (4) depending on regional differences in China. PPD constitutes to a prevalent mental disorder with clinical significance extending across the domains of psychology and gynecology (5). Its adverse consequences impact maternal well-being and common symptoms include anxiety, persistent fatigue, and enduring depressive states (6). PPD also affects the cognitive, behavioral, and emotional development of the newborns, with repercussions extending beyond adolescence (7). Historically, there is a lack of public awareness of mental illness and the prevalence of PPD has always been underestimated and poorly diagnosed particularly in developing countries (8). In China, traditional cultural values often contribute to the stigmatization of mental health issues, making it challenging to determine the overall mental health status of women living in China (9).

China’s rapid urbanization presents a unique context for studying PPD, particularly in megacities like Shenzhen has the fastest rates of growth (10). Shenzhen’s rise as an international city, fueled by significant local government investment, has created a distinctive socio-economic environment where 67.7% of the population comprises of young migrants with the average age of 32.5 who seek better job opportunities (11). This influx of a young, mobile population introduces specific challenges, including the separation from extended family and the absence of traditional family support where new mothers could rely on a close-knit family network for emotional and practical support during the postpartum period. In urban environments like Shenzhen, many families live in smaller, nuclear units, sometimes far from their extended relatives. This physical and emotional distance can lead to feelings of isolation and increased stress (12), particularly as new mothers navigate the demands of urban life without the immediate support they might have had in their home town. Thus, rural-to-urban migrants may have specific health care needs (13). These shifts may contribute to a different set of PPD risk factors compared to those in less urbanized or rural areas, where communal support structures are more prevalent. One particularly relevant factor is the experience of living with in-laws, who often come from rural areas with different educational backgrounds and lifestyles compared to their urban-dwelling daughters-in-law (14). The generational and cultural gap between urban women and their rural in-laws can introduce additional stressors, such as differing expectations regarding childcare, household responsibilities, and social norms (15). The lack of alignment in educational levels and familiarity with urban life may lead to conflicts, misunderstandings, and increased psychological pressure on new mothers, potentially exacerbating the risk of PPD. These dynamics are particularly pronounced in cities like Shenzhen, where rapid modernization often clashes with traditional values and practices brought by older generations.

Non-biological factors are related to psychological stressors (e.g., father abandonment, financial strain), the underlying cognitive vulnerabilities (e.g., negative attributional style) and the role of psychosocial resources (e.g., social support, self-esteem) (16). Globally, these risk factors can vary significantly depending on the cultural and socio-economic context. As an example, younger mothers (17) (<25 years old) were reported to be at higher risk of PPD, whereas in Sri Lanka, advanced maternal age (30–39 years old) is associated with higher PPD risk (18). Three primary categories of non-biological associated factors for PPD risk factors were identified in China, including prenatal emotion, social demographic factors such as poor marital relationship and lack of social support, and interpersonal factors (19). Considering the diverse cultural beliefs, social values, and localized socio-economic and environmental factors in different regions of China, it is likely that these elements could influence the incidence and characteristics of PPD. Risk factors for PPD can be categorized into biological and psychosocial risk factors. Additionally, Shenzhen’s status as a technology hub introduces new dimensions to maternal mental health, such as the role of social media and technology in shaping mothers’ experiences (20). The influence of online communities, exposure to unrealistic parenting standards, and the isolation that can accompany digital interactions are all modern stressors that may increase PPD risk (21) in this rapidly evolving city.

The aim of the present study investigated the living situation and mental health status of a cohort of puerperal women in Shenzhen to identify potential non-biological associated factors for PPD. The findings of the study extend the understanding of mental health issues in China and facilitate the development of local service provision. Findings of the study would also contribute to the global understanding of maternal mental health and may serve as a reference for other regions experiencing similar urbanization and cultural shifts.

## Methods

### Study population

Data collection took place between May and December 2019. A consecutive sampling method was adopted. All puerperal women who gave birth at Luohu Hospital of Maternal and Child Health Medical Center 6–7 weeks postpartum were invited to participate in this study. Women who met the inclusion criteria were included in the study. The study was approved by the Shenzhen Municipal Health Commission and Health Bureau of Luohu District, Shenzhen Municipal People’s Government (approval number: PJ2019102903). All participants provided written informed consent. The inclusion criteria were as follows: (1) aged between 18 and 40 years; (2) resided in Shenzhen for at least 1 year; (3) within 6 to 7 weeks postpartum; (4) first or second childbirth; and (5) no prior diagnosis of severe mental illnesses of anxiety disorder, bipolar disorder, schizophrenia, or obsessive-compulsive disorder. The exclusion criteria were as follows: (1) pregnancy-related medical complications such as pre-eclampsia, gestational hypertension, or severe gestational diabetes; (2) serious internal medical conditions, including cardiac diseases requiring long-term or intensive treatment; (3) on medications such as antidepressants or anti-anxiety drugs which may affect mental health assessment; and (4) individuals who were unable to understand the content of the interviews or cooperate with the researchers.

### Sample size calculation

The sample size calculation formula for estimating proportions in prevalence studies is derived from the principles of statistical estimation (22). This formula is commonly used in epidemiology and public health research to determine the sample size needed to estimate a population proportion with a specified level of confidence and margin of error. The formula is given by:


$$n=\frac{Z^{2}\cdot p\cdot \left(1-p\right)\;}{d^{2}}$$

where:

Z = 1.96 (Z-score for a 95% confidence interval);

*p* = 0.15 [15% estimated prevalence of PPD (3)];

*d* = 0.10 (desired margin of error).

Thus, a sample size of approximately 49 participants is needed to achieve a 10% margin of error with 95% confidence.

### Data collection

Data collection took place during the second month of postpartum. Participants completed a structured questionnaire through face-to-face interviews conducted by trained research assistants. Interviews were conducted in a private room to ensure participants felt secure and could provide honest and accurate responses. Each interview lasted approximately 60 to 90 min. Responses were securely recorded on encrypted filed and transferred to a password-protected database. Any discrepancies or missing data were promptly addressed by re-contacting participants when necessary.

### Postpartum depression

The Chinese version of the Edinburgh Post-natal Depression Scale (EPDS) questions was administered to assess postpartum depression. The Chinese translation of the EPDS was cross-validated through reverse translation into English to ensure equivalence with the original English version (23). The scale was previously demonstrated to have robust internal consistency (Cronbach’s α = 0.714) and test–retest reliability (Cronbach’s α = 0.814). Responses were scored on a scale ranging from 0 (not at all) to 3 (as much as I ever did). The total score ranging from 0 to 30, with higher scores indicating increased depression severity. Scores between 10 and 12 were indicative of minor PPD, while scores exceeding 13 were indicative of major PPD (24). Trained research assistants provided explanations to ensure adequate comprehension of each questionnaire section from each participants. When a participant had EPDS score above 9, our psychologist contacted that participant and re-administered the questionnaire 1 week later to verify the diagnosis of PPD. All women with confirmed PPD diagnosis were subsequently offered professional psychological consulting and treatments by psychologists.

### Questionnaires covariates

#### Occupations

Occupations were categorized into white-collar jobs, which involve office work or the application of specialized skills and knowledge such as management, law, and finance, and blue-collar jobs, which involve manual labor or technical work such as manufacturing, construction, maintenance, agriculture, and transportation. Considering the unique stability and security in terms of remuneration and benefits enjoyed by civil servants, administrative managers, teachers, and medical staff in China, this occupational group was segregated for analysis as “institution staff.” Additionally, self-employed individuals and freelancers, despite potential overlaps with white and blue-collar characteristics, were analyzed as a separate category.

#### Economic status

Economic status was determined using the *per capita* income of the household, calculated as the total household income divided by the number of family members living together. Utilizing the published average monthly wages of Shenzhen’s urban non-private and private sector employees in 2019 as a benchmark (25), “upper class” was defined as a *per capita* monthly income exceeding 1.5 times the average (i.e., above 12,241.58 CNY), “middle class” as between 0.75 and 1.5 times the average (i.e., between 6,120.79 CNY and 12,241.58 CNY), and “poverty level” as below 0.75 times the average (i.e., below 6,120.79 CNY).

#### Personality assessment

The personality traits of the participants were evaluated by a nationally certified psychological counselor. The Eysenck Personality Questionnaire (EPQ) was used to assess the extraversion/introversion dimension (26). The EPQ included questions addressing aspects such as talkativeness, number of friends, preferences for lively or crowded places, and enjoyment of being the center of attention. The Edinburgh Postnatal Depression Scale (EPDS) assessments were conducted subsequent to the personality evaluations to minimize potential researcher bias.

#### Age

The age of the participants was recorded in years.

#### Education level

Education levels were categorized into illiteracy, completed elementary school, completed vocational or technical training, higher education training, and postgraduate training based on participants self-reported category.

#### Feeding patterns

Feeding patterns were categorized into exclusive breastfeeding, combination feeding, and bottle feeding.

#### Antenatal depression and anxiety

The presence of antenatal depression and anxiety was assessed using standardized diagnostic criteria and self-reported measures.

#### Traumatic or stressful events during pregnancy

Participants were asked to report any traumatic or stressful events experienced during pregnancy.

#### Prenatal education

Participation in prenatal education programs was enquired.

#### Doula-assisted delivery

The presence of a doula during delivery was recorded.

#### Pain-managed childbirth

The use of pain management methods during childbirth was documented.

#### Newborn illness or disease

Any instances of newborn illness or disease were recorded.

### Statistics

All data were analyzed using SPSS 20.0 software. Descriptive analysis was conducted to assess the central tendency, dispersion, and frequency. Age was compared among groups using the Student’s *t*-test. Rank data were compared between groups using the Wilcoxon rank-sum test, and count data were compared using the chi-square test. Logistic regression analysis was employed to calculate the odds ratios (OR) and their corresponding 95% confidence intervals (CI) for all variables. Statistical significance level was set at *p* ≤ 0.05.

## Results

Table 1 shows the demographic data of the sample population. Data from 73 women with PPD and 430 women without PPD were included in the final analysis. Student’s *t*-test results indicated a statistically significant difference in the mean age of the groups: 29.57 years (±4.123 SD) for healthy mothers and 28.25 years (±3.785 SD) for PPD mothers (*p* = 0.01).

**Table 1:** Demographic characteristics of 503 participants.

Variables	Healthy controls (*N* = 430)	PPD patients (*N* = 73)	F for Chi-square	df	*p*
**Age (mean ± SD) (years)**	29.57 ± 4.123	28.25 ± 3.785	1.348	501	0.01
**Career**			2.26	4	0.688
Unemployment	197	33			
White-collar	139	28			
Blue-collar	10	1			
Institution stuff	34	6			
Individual businessman	50	5			
**Education**			0.865	4	0.93
Illiteracy	63	11			
Completed elementary school	51	10			
Completed vocational or technical training	82	11			
Higher education training	226	40			
Postgraduate training	8	1			
**Family**			7.092	2	0.029
Living with spouse only	186	20			
Living with parents	62	11			
Living with parents-in-laws	182	42			
**Economic status**			2.758	2	0.252
Upper class	102	11			
Middle class	321	61			
Poverty level	7	1			
**Feeding patterns**			5.222	2	0.073
Exclusive breast feeding	218	32			
Combination of both	117	29			
Feeding-bottle	35	12			
**Personality**			2.998	2	0.223
Extroverted	10	4			
Average	348	60			
Introverted	72	9			
**Antenatal depression**			39.602	2	0.000
Yes	24	20			
No	405	52			
**Antenatal anxiety**			64.283	3	0.000
Yes	46	35			
No	381	38			
**Traumatic or stressful event during pregnant**			14.978	1	0.001
Yes	14	10			
No	416	63			
**Prenatal education**			0.044	1	0.886
Yes	111	18			
No	319	55			
**Doula-assisted delivery**			0.04	1	0.841
Yes	113	20			
No	317	53			
**Pain-managed childbirth**			0.002	1	0.963
Yes	199	34			
No	231	39			
**Newborn illness or disease**			1.417	1	0.234
Yes	30	8			
No	400	65			

The results of Wilcoxon rank-sum test on the rank data indicated no significant differences in employment status (*p* = 0.688), educational levels (*p* = 0.93), and economic status (*p* = 0.252) between the two groups. For the count data, the results of chi-square tests indicated that age (*p* = 0.01), family living arrangement (*p* = 0.03), antenatal depression (*p* < 0.001) and anxiety (*p* < 0.001), and traumatic or stressful events during pregnancy (*p* = 0.00). No significantly difference was observed between the groups in career, level of education, economic status, feeding pattern, personality type, newborn illness, prenatal education, doula-assisted delivery, or pain-managed childbirth. Thus, the factors of age, family arrangement, depression or anxiety, and traumatic events are associated factors for PPD.

**Figure 1:**
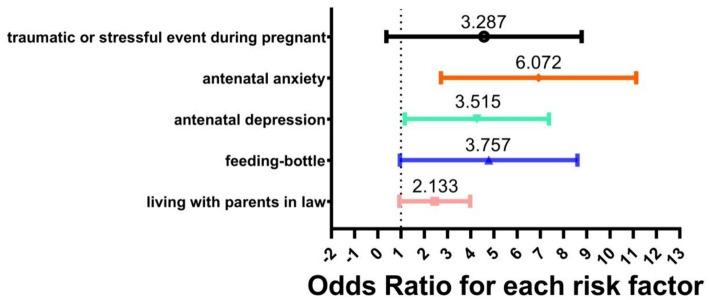
The odds ratio for each risk factor of PPD.

All factors that were significantly different between the two groups were subsequently included in binary logistic regression analysis. Table 2 shows the results of logistic regression analysis of all factors. Factors found to be significant predictors of PPD were: age (OR = 0.921, 95% CI: 0.864–0.981), living with in-laws (OR = 2.133, 95% CI: 1.108–4.106), exclusive bottle feeding (OR = 3.757, 95% CI: 1.567–9.006), prenatal depression (OR = 3.515, 95% CI: 1.61–7.675), prenatal anxiety (OR = 6.072, 95% CI: 3.209–11.49), and adverse life events during pregnancy (OR = 3.287, 95% CI: 1.165–9.269). Other factors were not found to have a significant effect. Figure 1 shows the Odds Ratio for each risk factor of PPD.

**Table 2:** Logistic regression analysis of contributing factors to PDD.

	B	S.E	Wald χ^2^	df	*p*	OR	Lower 95% CI	Higher 95% CI
**Age (years)**	−1.773	0.127	6.476	1	0.011*	0.921	0.864	0.981
**Career status**								
Unemployment	–	–	1.619	4	0.805	–	–	–
White-collar	0.195	0.379	0.265	1	0.607	1.215	0.578	2.555
Blue-collar	−0.497	1.147	0.188	1	0.665	0.609	0.064	5.759
Institution stuff	0.329	0.598	0.303	1	0.582	1.389	0.43	4.484
Individual businessman	−0.512	0.615	0.695	1	0.404	0.599	0.18	1.998
**Education level**								
Illiteracy	–	–	1.739	4	0.784	–	–	–
Completed elementary school	−0.03	0.584	0.003	1	0.960	0.971	0.309	3.05
Completed vocational or technical training	−0.414	0.554	0.558	1	0.455	0.661	0.223	1.958
Higher education training	−0.179	0.497	0.129	1	0.719	0.836	0.315	2.217
Postgraduate training	−1.649	1.477	1.245	1	0.264	0.192	0.011	3.479
**Family**								
Living with spouse only	–	–	5.228	2	0.073	–	–	–
Living with parents	0.594	0.453	1.721	1	0.190	1.812	0.746	4.403
Living with parents in-laws	0.758	0.334	5.138	1	0.023*	2.133	1.108	4.106
**Economic status**								
Upper class	–	–	2.324	2	0.313	–	–	–
Middle class	−0.603	0.396	2.316	1	0.128	0.547	0.252	1.19
Poverty	−0.039	1.272	0.001	1	0.975	0.962	0.08	11.625
**Feeding patterns**								
Exclusive breast feeding	–	–	11.245	2	0.004	–	–	–
Combination of both	−0.164	0.327	0.252	1	0.616	0.849	0.447	1.61
Feeding-bottle	1.324	0.446	8.804	1	0.003*	3.757	1.567	9.006
**Personality**								
Extroverted	–	–	0.725	2	0.696	–	–	–
Average	0.388	0.831	0.218	1	0.640	1.474	0.289	7.523
Introverted	0.296	0.434	0.465	1	0.495	1.345	0.574	3.149
**Antenatal depression**	1.257	0.398	9.958	1	0.002*	3.515	1.61	7.675
**Antenatal anxiety**	1.804	0.325	30.725	1	<0.001*	6.072	3.209	11.49
**Traumatic or stressful event during pregnant**	1.19	0.529	5.059	1	0.024*	3.287	1.165	9.269
**Prenatal education**	−0.401	0.359	1.248	1	0.264	0.67	0.332	1.353
**Doula-assisted delivery**	0.119	0.34	0.124	1	0.725	1.127	0.579	2.194
**Pain-managed childbirth**	−0.195	0.311	0.394	1	0.53	0.823	0.448	1.513
**Newborn illness or disease**	0.596	0.471	1.602	1	0.206	1.815	0.721	4.566

*Indicates statistical significance level of *P* <0.05 is reached.

## Discussion

The present study aimed to identify non-biological associated factors for PPD in Shenzhen city of China. The results indicated that age, living with parents-in-laws or the mothers’ parents and exclusive bottle feeding are the key non-biological associated factors for women who lived within the region.

Our data indicated that age was a significant non-biological associated factor for postpartum depression, with younger women being at a higher risk of PPD within the sample population. Maternal age is a common associated factor reported in the literature. However, there appears to be a lack of consistency on whether older or younger mother is at higher risk of PPD. A study conducted in Sweden suggested younger mothers (<25 years old) are at higher risk of PPD (17), while another study from Sri Lanka reported that advanced age (30–39 years old) (18) were associated with PPD. When compare to data collected from China, age was not found to be a significant risk factor for PPD (27, 28). The conflicting results may be related to the non-controlled recruitment process, where the sample population may not necessarily reflect a range of age groups that may have depression. Moreover, cultural differences also play a significant role in shaping the prevalence of PPD among different age groups. The perception of motherhood and the level of social support can vary across different cultures and may impact the mental health of mothers of different age groups (29). For example, study conducted in Canada reported that teen mothers received more support during pregnancy and after birth than adult mothers, despite equal likelihood to experience PPD if they received no support or minimal support after the birth of the baby (30). In the context of Shenzhen, a city marked by rapid urbanization and a migrant population, younger mothers might face unique challenges. These can include the pressures of adapting to urban life, managing the demands of a career while raising a child (31), and the potential of a lack of extended family support due to migration (32). Younger mothers from Shenzhen might feel pressured to conform to modern ideals of success and parenting, which can exacerbate feelings of inadequacy or stress, thereby increasing their risk of PPD. These pressures could be less pronounced for older mothers, who may have more established support systems and greater life experience to navigate the challenges of parenthood. Findings of the present study adds further support that age may not be a standalone associated factor of postpartum depression among women in Shenzhen. Further research that takes into consideration of age, cultural influences and social differences is recommended to better understand the relationship between age and postpartum depression and to develop targeted interventions program for the at-risk populations.

This study observed that the chance of postpartum depression is higher when the mother cohabits with parents-in-law. This observation aligns with published studies from China that identified poor relationship between mothers-in-law and daughters-in-law as a associated factor (19, 33). It was reported that the women usually felt more comfortable with their own parents who understood them and whom they found easier to talk to (11). When the relationship between mother and grandparents is conflictual or when grandparents interfere with parental childrearing, grandparents’ involvement may have negative effects on mothers’ affective state (34, 35). In China, conflicts between mothers-in-law and daughters-in-law are common (36). Traditionally, it’s expected that daughters-in-law show respect and adhere to the wishes of their elders (37). With the rise of more educated, modern-thinking women, these traditional practices are being questioned and new mothers are more likely to assert their beliefs in child-rearing. This clash of old and new perspectives may partly explains why living with in-laws may increase the likelihood of PPD. However, the latest meta-analytic evidence propose the association between support of the baby’s grandparental and better maternal mental health during the first year postpartum (38), indicating the potential benefit of living with the in-laws. The findings of our study contribute to expanding the understanding of non-biological associated factors, emphasizing the potential role of parental and in-law dynamics in the development of postpartum depression among women in Shenzhen.

Exclusive bottle feeding was observed as another associated factor for postpartum depression within this sample population from Shenzhen. Breastfeeding was widely reported in literature as a protective factor (39), with high level of anxiety and depression was associated with nonexclusive breastfeeding at 3 months postpartum (40). Stopping exclusive breastfeeding also reported to increase the likelihood for depression and anxiety (41). The hormone oxytocin released during early postpartum period due the need of breast feeding (42) has long been speculated to be inversely associated with postpartum depression but firm conclusion could not be drawn due to contradictory evidence and low quality of published trials (43). However, the association between bottle feeding and postpartum depression is not always straight forward with studies reported breastfeeding mothers are at increased associated of PPD (44, 45) or found no association (46, 47). The contradictory data may reflect the complex nature between socioeconomic factors such as maternal education, family income and marital status, quality of relationship and stressful life events, which ay all contribute to the occurrence of PPD. Urban environments like Shenzhen may put pressure on mothers to return to work sooner or may lack professional support for breastfeeding (48), leading to earlier adoption of bottle feeding. Many women may also feel obliged to breastfeed because of family or social expectations where breastfeeding is adopted as a strategy for them to be portrayed as calm, coping and in control when in reality they were struggling and not enjoying breastfeeding (49). Another factor to consider was the inability to breastfeed, rather than the mother’s personal choices to bottle feed. Thus, the combination of the intention of the mother to breastfeed and the capability to breast feed may be stronger influence on PPD occurrence (50). The findings of our study highlight the importance of considering breastfeeding practices as a potential protective factor against postpartum depression in the specific context of Shenzhen city. Further study is warranted to ascertain if the intention of mother to breastfeed who actually breastfeed may be a stronger protecting factor to PPD.

Like the vast majority of similar studies, we also find that the risk of postpartum depressive symptoms is higher when women experience anxiety or depression during pregnancy (2, 51) or when adverse life events occur, such as illness, domestic violence or arguments during pregnancy (52–54). It is important to acknowledge that anxiety or depression is not only caused by stressful events, but also by dramatic hormonal changes during pregnancy and childbirth that can trigger depression or anxiety symptoms. We cannot be certain that adverse events during pregnancy are the entire cause of mood disorders during pregnancy. Since all participants denied a previous history of psychiatric morbidity, it could not be ruled out that the stressful event they suffered during pregnancy were not a progression or continuation of an underlying psychiatric illness that began prior to pregnancy.

### Limitations

This study has several limitations that warrant consideration. First, the sample size is relatively small and the sample population was recruited from the same district in Shenzhen, which may limit generalizability, as the socioeconomic status, culture, and access to healthcare in this district may not represent the broader population. Second, the data collection relied on self-reported measures, which are subject to bias, including recall bias and social desirability bias. Participants may have underreported or over reported their symptoms or circumstances due to the stigma associated with mental health issues or the desire to present themselves in a favorable light.

## Conclusion

Our study identifies several non-biological associated factors for PPD in a developed city like Shenzhen in Southern China. Specifically, living with in-laws, exclusive bottle feeding, prenatal anxiety, depression, and adverse life events were found to significantly contribute to the development of PPD. These findings highlight the importance of considering a broad spectrum of psychosocial factors when addressing maternal mental health within this specific local region. As an example additional support for mothers living with in-laws may be required to promote mental health awareness during prenatal care, and offer stress management resources to those experiencing significant life events. Furthermore, public health initiatives might focus on educating families about the impact of feeding practices on maternal mental health and promoting support systems for breastfeeding. By addressing some of these identified factors, healthcare providers and policymakers can develop specific strategies to minimize the risk of PPD and improve overall maternal mental health in the region. Future research explore these relationships and evaluate the effectiveness of targeted interventions tailored to the local context.

## Data availability statement

The raw data supporting the conclusions of this article will be made available by the authors, without undue reservation.

## Ethics statement

The studies involving humans were approved by Shenzhen Municipal Health Commission and Health Bureau of Luohu District, Shenzhen Municipal People’s Government. The studies were conducted in accordance with the local legislation and institutional requirements. The participants provided their written informed consent to participate in this study. Written informed consent was obtained from the individual(s) for the publication of any potentially identifiable images or data included in this article.

## Author contributions

JH: Writing – review & editing, Writing – original draft, Project administration, Methodology, Investigation, Formal analysis. YL: Data curation, Formal analysis, Writing – review & editing, Writing – original draft, Methodology, Investigation. LC: Writing – review & editing, Writing – original draft, Supervision, Funding acquisition, Formal analysis. YZ: Writing – review & editing, Writing – original draft, Validation, Supervision, Methodology, Data curation, Conceptualization.
