# Corrigendum: Non-biological factors associated with postpartum depression among women in Shenzhen: a case-control study

**DOI:** 10.3389/fpubh.2024.1500641

**Published:** 2024-10-11

**Authors:** Jiangshan He, Yang Li, Ling Chen, Ying Zhang

**Affiliations:** ^1^Second Clinical College, Guangzhou University of Chinese Medicine, Guangzhou, China; ^2^Eighth Affiliated Hospital, Sun Yet-san University, Shenzhen, China; ^3^Maternity and Children Health Care Hospital of Luohu District, Shenzhen, China; ^4^National Clinical Research Center for Chinese Medicine Cardiology, Xiyuan Hospital, China Academy of Chinese Medical Sciences, Beijing, China

**Keywords:** postpartum depression, China, cultural factor, family relationship, depression

In the published article, there was an error in the order of the affiliations. “Second Clinical College, Guangzhou University of Chinese Medicine, Guangzhou, China” should be listed first for author Jiangshan He.

The authors apologize for this error and state that this does not change the scientific conclusions of the article in any way. The original article has been updated.

